# The prevalence of root canal treatment, periapical status, and coronal restorations in elderly patients in the Polish population

**DOI:** 10.1016/j.heliyon.2024.e35584

**Published:** 2024-08-21

**Authors:** Krystyna Pietrzycka, Mateusz Radwanski, Jukka P. Matinlinna, Monika Lukomska-Szymanska

**Affiliations:** a Department of Endodontics, Medical University of Lodz, 251 Pomorska Str., 92-213, Lodz, Poland; b Applied Dental Sciences, Biomaterials Science, Division of Dentistry, The University of Manchester, Manchester, M13 9PL, United Kingdom; c Department of General Dentistry, Medical University of Lodz, 251 Pomorska Str., 92-213, Lodz, Poland

**Keywords:** Cone beam computed tomography, Apical periodontitis, Root canal obturation, Treatment outcome, Epidemiology, Elderly population in Poland

## Abstract

**Objectives:**

The aim of this retrospective cohort study was to determine the prevalence and correlations between root canal treatment, periapical status, and coronal restoration detected using cone beam computed tomography (CBCT) in the elderly Polish population (60–79 years).

**Methods:**

A total of 480 CBCT images were assessed. Collected data included: age, gender, the tooth location, direct restoration, decay, single crown, abutment of fixed bridge, the quality of the restoration, root canal treatment, post and core, apical periodontitis (AP), and quality of root filling.

**Results:**

The number of teeth in the elderly patients decreased, while the number of endodontically treated teeth increased with age. More teeth were preserved in mandible, the most common group of teeth were incisors. AP was more often detected in the maxilla in general, and in mandibular molars. The over-filling was observed more frequently in maxilla, in maxillary molars and in mandibular premolars, while the short-filling in maxillary and mandibular molars. AP was significantly more often observed in short-filled root canals than in other length criteria (overfilled, adequately, lack of filling).

**Conclusions:**

Inadequate prosthetic restoration, presence of post, pulpotomy, missed canals and root canal treated teeth were associated with increased prevalence of AP.

**Clinical significance:**

The article indicates the need of treatment of elderly population in Poland. There is the demand to enhance the quality of endodontic and restorative treatment in this population. Additionally, CBCT examination, if indicated, should be implemented in the course of endodontic therapy to provide adequate information to clinicians.

## 1 Introduction

A growing trend in population ageing have been seen in recent years not only in Poland, *viz.* according to the prognosis of the World Health Organization (WHO) one in six people in the World population will be aged 60 years or over by the year 2030, and alone in Japan 30% of the population is already elderly [1]. According to Eurostat (2022) [2], the percentage of elderly population in the European Union amounted up to 21.1% and alone in Poland 25.7% [2]. The percentage of elderly people in the population of Poland and other countries is gradually increasing. At the end of 2022, the number of individuals aged over 60 reached 9.7 million and it is estimated to increase up to 10.8 million in 2030, and up to 13.7 million in 2050 [3]. Moreover, in 2050 elderly people (60 years and over) are expected to constitute about 40% of the total population of Poland [3], while in Australia it will be 21.6% [4] and 23% in the USA [5]. As the life expectancy extends, the desire to keep natural teeth also increases. Oral health conditions play an important role in the life quality of elderly people. Additionally, the relationship between general health and oral health status has been proven, especially in older adults [[6], [7], [8], [9], [10], [11], [12], [13], [14], [15]]. Therefore, it is of prior importance to maintain healthy dentition in elderly patients; as a consequence, treatment needs should be identified and recognized. Socioeconomic problems affect more elderly than younger population hindering even more their oral health status. Therefore, from a public health perspective, the oral health in elderly population should be known in order to monitor dental health needs and services.

Therefore, studies focused on elderly populations may help to disclose the needs for dental treatment and identify optimal treatment outcomes to prevent apical disease and edentulism. Many studies are based on OPG [[16], [17], [18], [19], [20], [21], [22], [23], [24], [25], [26], [27], [28], [29]] or periapical radiographs [18,24,[30], [31], [32], [33], [34], [35], [36]], however these methods have limitations, such as poor quality of diagnosis [[37], [38], [39]]. On the contrary, cone beam computed tomography (CBCT) [[40], [41], [42]] imaging provides essential information on the anatomy, such as the number and configuration of root canals, the status of the tooth (direct restoration, decay, prosthodontic restorations, and endodontic treatment) and the periapical tissues.

Many epidemiological studies have used scoring systems for radiographic assessment of apical periodontitis (AP). The periapical index (PAI) [43] and criteria suggested by de Moor [44] were employed to evaluate AP in OPG or periapical radiographs. PAI is a 5-point ordinal scale, where PAI 1–2 is considered as ‘healthy’ and PAI 3–5 as ‘diseased’ [[17], [18], [19],[22], [23], [24], [25], [26],^29^,[32], [33], [34], [35], [36],[45], [46], [47], [48], [49]]. The criteria proposed by de Moor [44] for periapical tissue status are as follows: 1) the apical periodontitis represents widening of the periodontal ligament, *i.e.,* widening of the apical part of the periodontal ligament not exceeding 2 times the width of the lateral periodontal ligament space, or, 2) periapical radiolucency, *i.e.,* connected the apical part of the root, exceeding 2 times the width of the lateral part of the periodontal ligament [19,27,50,51]. With advances of technology, CBCT and respective the Cone Beam Computed Tomography Periapical Index (CBCTPAI) [52] were introduced [53]. Hence there is still an insufficient number of studies using this method, especially to determine endodontic treatment needs in elderly population in Poland.

This study focuses on oral health condition in an elderly population. In Poland patients have access to both the public and private dental care, but the percentage of elderly patients receiving regular dental treatment remains unknown.The aim of this retrospective cohort study was to determine the prevalence and correlations between root canal treatment, periapical status, and coronal restoration detected using cone beam computed tomography in elderly Polish population. The null hypothesis is that there are no differences in the prevalence of root canal treatment, periapical status, and coronal restorations in evaluated groups.

## 2 Materials and methods

### 2.1 Sample selection

The CBCT scans were randomly selected from the pool of the accessible examination taken between 2018 and 2022. CBCT images obtained from 480 patients reported to private dental practice and the Radiology Department (Central Clinical Hospital, Institute of Dentistry, Medical University of Lodz, Poland) were analysed in this study. The inclusion criteria concerned patients aged 60–79 were: at least one tooth present, good quality CBCT scans showing upper and lower jaw including the roots, and periapical areas of all teeth. The exclusion criteria of the study comprised: edentate patients aged 60–79, patients aged up to 59 and over 80, CBCT scans with no teeth, low quality or with additional artefacts, or images only of maxilla or mandible.

All CBCT images were performed using the GX CB-500 device (Gendex Dental Systems, Hatfield, PA, USA) at 120 kV and 5.0 mA, with a voxel size 0.125–0.25 mm and an exposure time of 20 s. All images were analysed using specialized computer software (iCATVision Q, ver. 1.9.3.13; Gendex, USA), or Kodak 9500 3D system (Carestream Health, Marne-la-Vallée, France) at 10 mA and 90 kV, a medium field of view of 9 cm × 15 cm, and a 0.2 mm voxel size.

No patient underwent a CBCT examination exclusively for this study. All patients' data were anonymized; only the gender and age at the time of performing the CBCT examination were recorded.

The sample size calculator was used to calculate sample size. The Polish aged 60 years and over population is about 9.8 million people. The recommended sample size with an error margin of 5% and a confidence level of 95% was 385, based on Sample Size Calculator [54].

The Ethics Committee of the Medical University of Lodz has approved this research (RNN/333/15/KE).

### 2.2 CBCT evaluation

The CBCT images were analysed in the MSI WindTop AE2220, LCD 21.5-inch screen with a resolution of 1920 x 1080 pixels, full-HD in a darkroom. CBCT images were oriented as Bürklein et al. proposed [41]. The brightness, contrast, and sharpness of all the images were adjusted using the image-processing tool in the software to provide optimal visualization. The magnifying tool was also used. All samples were manually evaluated by two independent observers, endodontists (K.P., M.R.) who had previously qualified and experienced in CBCT imaging. The calibration was performed on 30 cases. In the event of disagreement, the case was discussed until a consensus was reached. The judgement of teeth and periapical tissue was performed using view Multiplanar Reconstruction (MPR) of the manufacturer's software viewer (iCATVision Q, ver. 1.9.3.13; Gendex, USA; Carestream Health, Marne-la-Vallée, France). The axial, coronal, and sagittal slices analyses for each tooth were performed. On the axial, sagittal, and coronal slices, vertical and horizontal lines parallel to the long axis of each tooth's root were aligned. When necessary, in order to improve the quality of the image, it was allowed to use the software by changing contrast, brightness or using magnification tools.

### 2.3 Tooth status

Data for each tooth were recorded and included: the location, direct restoration type (amalgam, glass-ionomer, or composite resin filling), decay, single crown, abutment of fixed bridge, root canal treatment, post and core, periapical status (Table 1). A direct restoration was considered as a restoration of the coronal part of the tooth. The quality of the restoration was also assessed.

**Table 1:** Parameters assessed in the study.

	Criteria	Definition
**Coronal structure**	*Tooth without restoration*	
	Sound	Tooth without caries and restoration
	Primary caries	Appearance of coronal radiolucency
	*The status of direct restorations*	
	Direct restoration	Restoration of the coronal part with contrast corresponding to amalgam, glass-ionomer or composite resin filling
	Inadequate direct restoration	Absence of restoration or/and caries
	*The status of indirect/prosthodontic restorations*	
	Crown	Coronal restoration of the tooth with appearance of prosthetic restoration
	Bridge abutment	Tooth supporting the prosthetic bridge
	Inadequate prosthetic restoration	Crown or bridge abutment with overhangs, open margins or caries adjacent to the prosthetic restoration
	Post: metal/non-metal	Cast restoration with radiographic opacity in crown and canal simultaneously
	*Status of endodontic treatment*	
**Endodontic structure**	Endodontically treated tooth	Radiopaque material in the pulp chamber and/or root canals
	Previously initiated root canal treatment	Radiopaque material only in the pulp chamber in single/multi-canal tooth
	Missed canal	Absence of any contrast material in the canal when other canal(s) were filled (in multi-canal tooth)

### 2.4 The quality of root filling

The quality of root filling was evaluated according to the length (Table 2). The disadvantages of CBCT images are artefacts caused by dense materials in root canals (such as gutta-percha and sealers and/or other hyperdense materials), which are producing nonuniformities in grey level. The mentioned artefacts can affect the image quality and the anatomic accuracy. The most common artefacts appear as different patterns such as a cupping artefact, hypodense halo, streaks, and dark bands that are created by the phenomenon called beam hardening [[55], [56], [57], [58]]. However, due to different artefacts, homogeneity was not evaluated.

**Table 2:** Evaluation of length of root canal filling.

Length criteria	Definition
**Adequate**	Root filling ≤2 mm from radiographic apex
**Overfilling**	Root filling beyond the radiographic apex (gutta-percha cones or/and sealer)
**Short filling**	Root filling >2 mm from radiographic apex
**Lack of filling**	Absence of any opaque material

### 2.5 Cone Beam Computed Tomography Periapical Index

AP was evaluated using CBCTPAI. It is a 6-point (0–5) scoring system with two variables: expansion of cortical bone (E) and destruction of cortical bone (D) (Table 3) [52]. AP was defined as a radiolucency connected with the apical part of the root, score: 1–5, E, D according CBCTPAI. Multirooted teeth were classified according to the most severe score of periapical condition. The prevalence of AP on the tooth level was defined as the percentage of AP out of the total number of examined teeth (n = 8226). The prevalence of AP on the subject level was defined as the percentage of patients with at least one tooth with AP out of the total number of patients (n = 480).

**Table 3:** Evaluation of The Cone Beam Computed Tomography Periapical Index (CBCTPAI): scoring 0–5, E and D.

Score	Definition
**0**	Intact periapical bone structures
**1**	Diameter of periapical radiolucency >0.5–1 mm
**2**	Diameter of periapical radiolucency > 1–2 mm
**3**	Diameter of periapical radiolucency > 2–4 mm
**4**	Diameter of periapical radiolucency > 4–8 mm
**5**	Diameter of periapical radiolucency >8 mm
**E**	Expansion of periapical cortical bone
**D**	Destruction of periapical cortical bone

### 2.6 Statistical analysis

All statistical analyses were performed with the statistical software package Statistica 13.1 (StatSoft, OK, USA). The strength of linear relationship between two variables was measured with use of the Pearson correlation. Chi-square tests were used to compare the qualitative variables. MedCalc for Windows, version 12.5 (MedCalc Software, Ostend, Belgium) (https://www.medcalc.org/calc/odds_ratio.php), was used to calculate z-test for determination of association between variables. In all cases, statistical significance was considered at p < 0.05.

## 3 Results

The kappa score for interexaminer agreement after the first radiographic evaluation was 0.8, and the score for intraexaminer agreement was 0.9 after the second radiographic evaluation performed 1 week later, both indicating good agreement [59]. Results regarding characteristics of study group along with correlation between gender and age of patients and apical periodontitis were presented in Supplementary files 1 and 2.

### 3.1 Number of preserved teeth

The average number of teeth in the study population amounted up to 17.14 teeth (1–32 teeth). The average number of teeth among women (17.38 teeth) was higher than among men (16.85 teeth), however the difference was not statistically significant (p > 0.05).

**Fig. 1:**
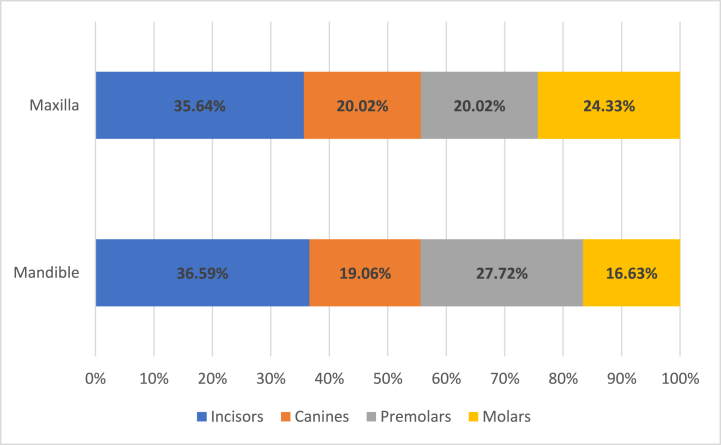
The distribution of preserved group of teeth in relation to dental arch.

Additionally, more teeth were preserved in the mandible (average 9.26 teeth) than in the maxilla (average 7.88 teeth) (p < 0.05). The most common presented group of teeth in both the maxilla and mandible were the incisors (p < 0.05). Molars were more frequently preserved in the maxilla than in the mandible (p < 0.05). On the other hand, in the mandible compared to the maxilla, premolars were present more often (p < 0.05) (Fig. 1).

### 3.2 Correlation between age of patients and number of teeth

**Fig. 2:**
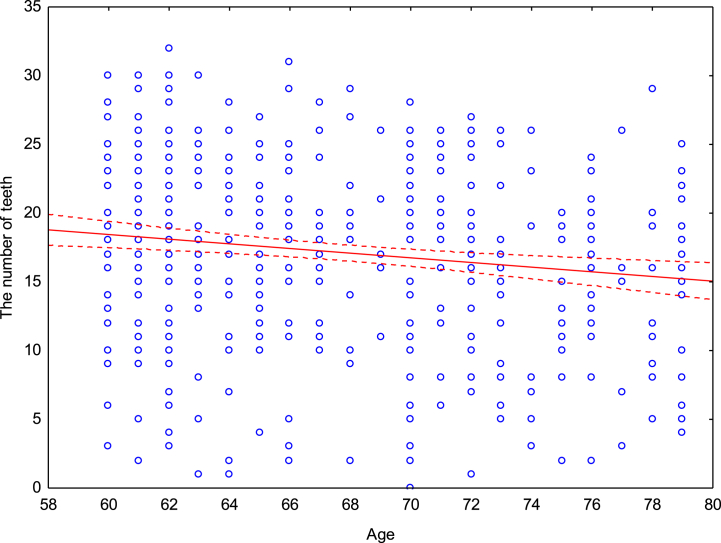
The relationship between the age of patients and the number of preserved teeth.

There was a statistically significant correlation between the age of the patients and the number of preserved teeth (r = −0.1525; p < 0.05) (Fig. 2). With age, the number of teeth in patients decreased (60–69 years - 17.92 teeth, 70 years and more - 16.07 teeth).

### 3.3 Teeth without restorations

Sound teeth (without fillings, caries, RCT or PAI0) were found in 26.67% of all teeth examined. Moreover, patients having all healthy teeth comprised 8.33% of the investigated population. The average number of sound teeth amounted up to 4.57 teeth. The prevalence of teeth with caries was found in 6.2% of all teeth examined.

### 3.4 The status of direct restorations

The number of teeth with direct restoration and inadequate direct restoration of the analysed teeth were found in 41.84% and 4.24%, respectively.

### 3.5 Correlation between the status of coronal structure and apical periodontitis

There was found no statistically significant correlation between the teeth with caries, direct restoration, inadequate direct restoration, and apical periodontitis. The distribution of coronal features of the teeth and apical periodontitis is presented in Table 4.

**Table 4:** The distribution coronal features of the teeth and apical periodontitis.

**Periapical status**				
	Normal (*n*)	AP (*n*)	Total (*n*)	p-value
**Caries**	324 (50.86%)	313 (49.14%)	637	p > 0.05
**Direct restoration**	2607 (75.74%)	835 (24.26%)	3442	p > 0.05
**Inadequate direct restoration**	198 (56.73%)	151 (43.27%)	349	p > 0.05
**Inadequate prosthetic restoration**	16 (28.07%)	41 (71.93%)	57	p < 0.05

### 3.6 The quality of fixed prosthodontic restorations

Inadequate prosthetic restoration was found in 0.69% of the analysed teeth (Table 4).

### 3.7 Correlation between the quality of fixed prosthodontic restoration and apical periodontitis

A crown with overhangs, open margins or caries adjacent to restoration (inadequate prosthetic restoration) was associated with an increased prevalence of AP (p < 0.05).

### 3.8 Correlation between the type of prosthodontic restoration and apical periodontitis

The type of prosthetic restoration (a single crown *vs*. bridge abutment) was associated with the apical periodontitis (p < 0.05) (Table 5). Lesions were found significantly more frequently in the bridge abutment teeth than in single crown abutments (p < 0.05).

**Table 5:** Distribution of periapical periodontitis lesion according to tooth restorations.

		Periapical status			
		Normal (n)	AP (n)	Total (n)	p-value
**Teeth with prosthetic restoration**	Single crown	550 (51.94%)	509 (48.06%)	1059	p < 0.05
	Abutment of bridge	468 (45.39%)	563 (54.61%)	1031	
**Teeth without prosthetic restoration**		5038 (82.11%)	1098 (17.89%)	6136	p > 0.05
**Total**		6056 (76.32%)	2170 (26.38%)	8226	

Moreover, the presence of a post metal/non-metal was significantly associated with the periapical lesion (z = 22.216 p < 0.001, 95% CI) as shown in Table 6.

**Table 6:** Correlation between periapical status and presence of post.

Periapical status				
	Normal (*n*)	AP (*n*)	Total (*n*)	p-value
Teeth with post metal/non-metal	456 (43.59%)	590 (56.41%)	1046	p < 0.001
Teeth without post metal/non-metal	5600 (77.99%)	1580 (22.01%)	7180	p > 0.05
**Total**	6056 (76.32%)	2170 (26.38%)	8226	

### 3.9 The status of endodontic structures

A total of 8226 teeth was evaluated, and 22.56% of teeth was endodontically treated. A total of 89.17% of patients had one or more teeth endodontically treated. Pulpotomy was performed in 1.89% of endodontically treated teeth. If the radiopaque material only in the pulp chamber in single/multi-canal tooth was noticed, AP was detected significantly more frequently (p < 0.05). A total of 12.77% of treated canals was missed, significantly more commonly in molars (p < 0.05) and in the maxilla than the mandible (p < 0.05).

### 3.10 The length of root filling

The overfilling of the material (sealer or gutta-percha) was found in 7.17% cases, significantly more often in the maxilla than in the mandible (p < 0.05). Overfilling most commonly was found in the molars in the maxilla and the premolars in the mandible (p < 0.05). A total of 50.89% (the maxilla) and 57.23% (the mandible), making an average 53.25% of treated canals, were filled clinically adequately. The short-filling (39.58%), both in the maxilla and the mandible, was found significantly most often in molars (p < 0.05).

### 3.11 Periapical lesions

**Fig. 3:**
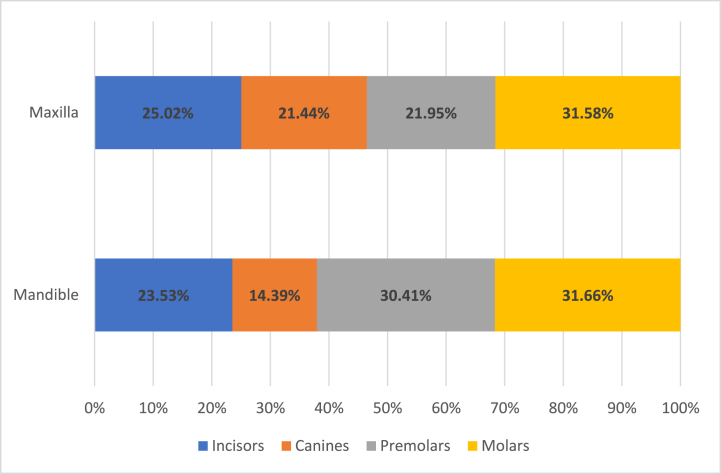
The distribution of apical periodontitis (AP) in relation to dental arch.

Periapical lesions were found more often in root canal treated teeth compared to vital teeth (p < 0.05). The periapical lesions were statistically more often detected in the maxilla in general and in mandibular molars (p < 0.05). Comparing the distribution of apical periodontitis between dental arches, in the maxilla, incisors and canines were more frequently affected, while in the mandible - premolars (p < 0.05) (Fig. 3).

The distribution of PAI score is presented in Table 7. Most of the apical lesions had the dimensions of 0.5–1 mm, followed by 1–2 mm. Moreover, the periapical cortical bone was not affected in 73.27% of all evaluated cases.

**Table 7:** Distribution of CBCTPAI score in examined population (E-expansion of cortical bone, D-destruction of cortical bone).

CBCTPAI															
**PAI score**	0	1	2	E	D	3	E	D	4	E	D	5	E	D	Total
Number of teeth and their %	6056 (73.62%)	1113 (13.53%)	587 (7.14%)	1	8	310 (3.77%)	6	69	83 (1.01%)	2	27	77 (0.94%)	18	45	8226

The presence of periapical lesions was reported in 91.67% of patients. In the evaluated population, an average of 3.87 teeth had periapical lesions and an average of 4.52 teeth were endodontically treated. The frequency of endodontic treatment significantly increased with the age of patients (r = 0.0277, p < 0.05). The average number of treated teeth in the 60-69-year-old individuals amounted up to 4.35 teeth, and in the 70-79-year-old ones 4.38 teeth.

### 3.12 Correlation between the root filling and apical periodontitis

The presence of root filling was significantly associated with the periapical lesion (z = 39.002, p < 0.001, 95% CI), shown in Table 8. Periapical lesions were significantly most often observed in the case of short-filled root canals (42%) compared with other length criteria (overfilled, adequately, lack of filling) (p < 0.05). If the root canal was missed and left unprepared during endodontic treatment, periapical lesions were found significantly more often than in case of teeth in which all canals were prepared and adequately obturated (p < 0.05).

**Table 8:** Correlation between periapical status and presence of root filling.

Periapical status				
	Normal (n)	AP (n)	Total (n)	p-value
**Teeth with root filling**	652 (35.13%)	1204 (64.87%)	1856	p < 0.001
**Teeth without root filling**	5404 (84.84%)	966 (15.16%)	6370	p > 0.05
**Total**	6056 (73.62%)	2170 (26.38%)	8226	

## 4 Discussion

The prevalence of AP and root canal treated teeth has been vastly investigated globally in general population [18,19,26,28,32,[60], [61], [62], [63], [64], [65], [66]]. In the literature factors, such as the distribution of teeth with caries [33,40,[66], [67], [68]], direct [16,40,[66], [67], [68]], prosthetic restoration [29,33,40,51,[66], [67], [68], [69]], or restorations using posts [29,47,51,68,70], the number of preserved teeth, the prevalence of root canal treatment, periapical status in both adult (OPG and CBCT) and elderly populations (OPG) have been also estimated and presented [8,9,12,14,15,18,19,21,24,26,28,32,40,[44], [45], [46],^50^,^64^,^67^,[71], [72], [73], [74], [75], [76], [77], [78], [79]]. Hence, there are only few studies that concentrated on the elderly population [24,30,33,35,62,80]. The present paper evaluated a total of 8226 teeth of 480 elderly patients (Polish population) focusing on the prevalence of root canal treatment, periapical status, and coronal restorations. It is noteworthy that to date this is the first study investigating these parameters in Polish population aged over 60. The null hypothesis was rejected because significant differences were found between compared parameters.

### 4.1 Study population group

The sample size of the present survey (480 individuals) is higher compared with that of some other studies (16–450) [16,18,20,[27], [28], [29],^32^,^34^,^35^,^68^] and, on the other hand, lower than in some others (487–884) [25,63,67,81]. In the present study and some other epidemiological studies, more women were included than men [29,30,[32], [33], [34],^40^,^41^,^61^,^66^,^67^,^82^,^83^] or only women were clinically evaluated [81]. Whereas, others included more men [18,25,35,65,68] or both genders in equal proportions [27,28].

The average age of the investigated population in present study amounted up to 67.72 years, similarly to a previous report [16]. Some other studies examined older (over 81 years) [20,80] or younger (52–66 years) population cohorts [30,35,36].

The correlation between the gender and age of patients and apical periodontitis, number of preserved teeth and the correlation between age of patients and number of teeth were discussed in Supplementary files 3, 4 and 5.

### 4.2 The status of coronal structure

The current paper estimated the coronal status of elderly's teeth, which was examined in some previous studies [16,18,40,68,81]. The prevalence of teeth with caries in the literature was in the range from 1.45% to 20% [33,40,[66], [67], [68]], which is in accordance with the current report and its findings (6.2%). In the present study number of teeth with direct restoration (41.84%) was similar [16,66,67] or higher than in other studies (29.3%) [40,68]. A possible reason for these differences could lie in the characteristics of sample used in the latter study, *viz.* the quantity (1160 patients) and the average age of patients, 48.4 years. The present research revealed inadequate restorations in 4.93% (inadequate: direct restoration 4.24%, prosthodontic restoration 0.69%), which is in conflict with findings from other reports (44%) [66]. The reason for this discrepancy could be probably in the methodology, namely in the latter study a lower number of teeth (1086 *vs.* 8226) and periapical radiographs were assessed.

In the current paper sound teeth (without fillings, caries, root canal treatment, and periapical lesions) (26.67% teeth) were diagnosed in 8.33% of patients. Contrasting data were found for the Greek and Estonian adult populations, where 52.7–52.8% of teeth were intact [33,67]. Moreover, in the investigated population the average number of sound teeth (without caries, restoration, endodontic treatment, and/or AP) amounted up to 4.57 teeth per person. A significantly higher value was found for the Estonian adult population, 14.6 teeth [67]. Discrepancies between this and abovementioned studies probably were related with different age range, *viz.* 60–80 years in this study *vs.* 16–77 years for the Greek and 35.5 ± 19.2 years for Estonian population, and a differentiated definition of tooth status, namely, ‘intact’ defined as no radiographic signs of caries or restoration, with no information about periapical status.

### 4.3 Correlation between the incident of caries and status of direct restoration and apical periodontitis

This correlation was not statistically significant. This result corresponds with other [86], which, on the other hand, is not supported by others [18,33,40,63,66,67]. In the current study AP was not significantly associated with the inadequate direct restoration, parallelly to some others [18,40,86].

### 4.4 The quality of fixed prosthetic restoration

Prosthetic restoration (a crown and bridge) was in 25.4% cases of this study, which is similarly in the Estonian population [67]. Contrasting data were presented by others, namely, higher (40%–94.73%) with only endodontically treated teeth were evaluated [29,33,51,66], or a lower incidence (2.59–14.6%) of prosthetic restorations [16,33,40,68,69].

Interestingly, inadequate prosthetic restoration (marginal gap or overhang) was found in 2.72% of crowned teeth, hence higher values were found in the literature (15.94–64.51%) accessing only teeth with RCT [29,33,69]. Moreover, in the abovementioned studies [29,33,69] inadequate restorations was defined as inadequate direct restoration and prosthetic restoration. In current study post metal/non-metal and core were observed in 12.71% teeth, hence higher (31.8–53.76%) prevalence of posts was determined using OPGs [29], and CBCT scans [51]. In contrast, significantly lower values were also reported (1.14%), however, these authors evaluated only teeth with AP [68].

### 4.5 Correlation between the quality of fixed prosthodontic restoration and apical periodontitis

In the present study an inadequate prosthetic restoration (marginal gap or overhang) was associated with AP, and this is supported by others [67]. According to the literature, teeth with crown restorations (with or without post/core) were more likely to have periapical radiolucency [16,18,63,79], which was confirmed in this study, where the bridge abutment and the presence of a post metal/non-metal was correlated with AP. However, it was not supported by the recent study [87].

Hence, one study reported that the relationship between AP and quality of coronal restoration was not statistically significant [86]. This difference could result from a different study protocol: a smaller sample size and only root canal treated teeth were evaluated in the above-mentioned study.

### 4.6 Correlation between the type of prosthodontic restoration and apical periodontitis

In the literature the prosthetic restoration (crown and bridge) was related with the AP [16,18,53,63,68,69,88]. Similarly to earlier papers [16,40]*,* in the present paper AP was found more often in teeth restored with a crown (or bridge). This is in contrast with a previous study [86], where teeth without crowns presented a higher prevalence of AP than crowned ones. These divergences with the latter report [86] could be attributed to the differences in methodology – only teeth with RCT were evaluated in the abovementioned study, and also the samples size (2090 *vs.* 199). Contrasting data were published by others, where the relationship between AP and type and quality of coronal structure was not statistically significant [86]. Interestingly, a lower risk of AP if the tooth was restored with a crown and bridge abutment was noticed in the Estonian report [67]. It was in contrast to the current study, among the type of prosthetic restoration, lesions were found more frequently in bridge abutment teeth than in crowns. Alike others [32,78], in this study an inadequate prosthetic restoration was associated with an increased prevalence of AP.

Periapical disease was significantly associated with an intracanal post which was confirmed in the literature [34,47,53,66,68,[89], [90], [91]] and the present study, which is in conflict with others [66,67,[92], [93], [94]]. Contrasting data were published, where placement of a post would not *per se* decrease the probability of periapical healing [70]. Additionally, teeth restored with posts exhibited a higher prevalence of AP compared with those restored with use of composite resin, or amalgams [34,41,42,53,95]. The key factor decreasing the probability of periapical healing is the inadequacy of the filling of the root length [96] in contrast to the placement of a post [70].

### 4.7 The length of root filling

The prevalence of teeth with RCT was discussed in Supplementary file 6.

An acceptable length of root canal filling was found in 53.26%, similarly to previous studies [61,66,97], where OPG was evaluated. Hence, the higher percentage, 61.9–65.35%, was found in some CBCT studies [51,69,86] in the adult population. These higher values could result from Refs. [51,86] discrepancies in the age of participants (15–72 years), or the group of examined teeth - maxillary molars were excluded from the study [69]. Interestingly, a lower prevalence of the adequate length of root canal filling was observed in the Austrian (20.8%) [29] and Estonian population (29.41%) [67], and Turkish subpopulation (41.87%) [22]. These differences in results could be due to a variable age range (16–91 years *vs.* 60–79 years), a higher number of teeth and type of radiographic examination (OPG *vs.* CBCT).

Overfilling in the current research was found in 7.17% of cases, which was in accordance with other research [16,66,99]. Hence, other studies reported lower (0.3–6.6%) [16,29,51,66,67] or higher values (9–32.83%) [35,41,68,69,86]. On the contrary, in the present study short-filling was determined in 39.58% of root canal treated teeth, which was in agreement with previous results [41,51,66,68] and in contrast with earlier reports (3.73–5.0%) [35,69]. In the latter study [69], a lower number of teeth (234 vs 8226) and younger patients (median: 48 years) were assessed. In the other paper [35] although similar sample of 65-year-olds individuals was evaluated, periapical radiographs were estimated. However, higher values were found in the American elderly subjects (52.07%) [16], Turkish adults 58.13% [22], Portuguese adult population (72.7%) [99] and in an Austrian subpopulation (75.4%) [29]. This might be attributed to the lower effectiveness of the OPG (used in American and Turkish population) in the assessment of RCT than CBCT. In a previous study [69] many short- or over-fillings on periapical radiographs were diagnosed as exhibiting a proper length on CBCT.

Radiopaque material only in the pulp chamber in single/multi-canal tooth was noticed in 1.89% endodontically treated teeth, similar percentage was reported other studies from Poland (1.4%) [27], Greece (1.3%) [33], the USA (1.77%) [16], and Estonia (5.2%) [67].

The prevalence of missing canals during shaping (12.77%) in the current study was similar to previously reported (12%) [100], and higher than in other papers (1.3–8.4%) [40,41,86]. This dissimilarity could be explained by the different range of age (15–72 years *vs.* 60–79 years). In elderly patients aging change, such as the pulp space diminishing and root canals calcification, are more often seen than in younger subjects [101]. Missed canals were most common in molars, significantly more often in the maxilla than the mandible, which finding was previously presented [41,100,102]. The presence of missed canals in maxillary molars could be associated with complex anatomy, i.e. the second mesiobuccal canal (in the mesiobuccal root) and difficulty in preparing a straight-line access cavity, with lack of pre- or intraoperative CBCT and with treatment without magnification.

### 4.8 Periapical lesions

The prevalence of AP in subjects in the current study was higher (91.67%) than in earlier reports, where in an adult population the amounts were from 25.2% to 83.7% (diagnosed with CBCT and OPG) [26,29,31,49,50,53,68,72,75,76,98], while in elderly individuals they range from 16% to 85.6% when diagnosed with OPG [16,17,19,25,29,35,45,67,68,77,84,98].

In the current study the prevalence of AP on the tooth level was 26.38%; similar data were earlier presented [18]. That said, some contrasting data in elderly population were presented in literature [16,24,32,35,45,73,75,85], lower (2.9–13.3%) [16,24,32,35,41,42,68,73,75,85,98,99]*,* and higher (42%) [45]. It is likely that these discrepancies could be related to the diagnostic method (full mouth X-ray series or OPG) and the lower sample size (32–450 *vs.* 480) and patients in the more differentiated age (12–85 *vs.* 60–79) were evaluated. In this study, an average of 3.87 teeth (per one patient) had AP and an average of 4.52 teeth were endodontically treated. Slightly lower values were observed in another study: 1.13 teeth with AP; 3.57 with root canal filing [24]. A relatively high number of endodontically treated teeth in Polish elderly population is a proof of accessibility of this treatment and social needs. Hence, a relatively high number of teeth with AP reflects the demand for high quality endodontic treatment.

In this research the AP were statistically the most often detected in the maxilla which is in agreement with previous studies [40,41,51]. Moreover, others reported (using CBCT) no difference between the incidence of AP in upper and lower teeth [66,98]. This may be attributable to the difficulty in gaining access to the canals of upper teeth.

Regarding the AP location in the maxilla, incisors and canines were more frequently affected, similar results were observed in adult Turkish subpopulation [86]. Similar result were found in the adult Jordanian subpopulation; AP was more often diagnosed in upper molars and premolars [50].

In this research the AP the most often affected mandibular molars, which is in agreement with previous studies [33,67,86,98], and this may be attributable to the complex anatomy of these teeth, and the difficulty in proper cleaning and shaping and filling during RCT of multi-canal teeth. However, other researchers obtained ambiguous results [35]. The discrepancies between the present and the abovementioned study resulted from a lower number of subjects (450), and more teeth were investigated (11484 *vs*. 8226) but on the periapical radiographs.

Moreover, in the current study APs were found more often in root canal treated (55.48%) compared to vital teeth (44.52%) which finding was confirmed in previous papers [25,28,32,33,40,67,76,97], and which was in contrast with others [77,96]. According to the literature, the percentage of AP in RCT teeth in older people is lower (14–42.5%) [16,20,36,73,75,85]. This difference could be due to the discrepancies in the number of evaluated teeth per individuals with RCT, type of radiographic examination (OPG, periapical radiographs, CBCT), and estimation method of AP (PAI, PAICBCT). CBCT scans, due to their greater sensitivity and specificity allow to the accurate assessment of status of periapical health and improve the reliability of prevalence of AP [52]. For this reason, it is used as an indicator of the periapical tissue condition in epidemiological studies. CBCT PAI based on the interpretation of CBCT scans has been used in numerous previous studies [98,[103], [104], [105], [106], [107]].

The prevalence of AP in teeth with RCT in this study was high (64.87%), similarly to other studies (60.0–61.5%) [33,86]. On the contrary, others reported lower values: 35.5–51.9% [16,22,32,66,76]. Reasons for this disagreement could be the methodology of the cited study: a lower sample size, different diagnostic method (full-mouth radiographic survey), definition of healthy periradicular status (the contour and width of the periodontal ligament space were normal, and the appearance of the surrounding bone was normal). Interestingly, a higher prevalence of AP in RCT, than in the current study, was observed in an urban Iraqi adult subpopulation (80.2%) [76]. The differences in results of latter study could be attributed to the discrepancies of methodology, lower number of CBCT scans (385), and significantly younger participants (18–45 years).

Considering the size of AP, most of them exhibited the diameter of 0.5–1 mm (CBCT-PAI score 1), followed by 1–2 mm (CBCT-PAI score 2), CBCT-PAI score 3 was seen twice less often than score 2. In contrast, it is noteworthy that others reported that CBCT-PAI score 4 and 1 was observed in the majority of the lesions [98]. A significant consideration of the current study is that periapical cortical bone was not affected in 73.27% of all cases, what was in agreement with earlier study (76.2%) [98]. In addition to that, the prevalence of the periapical cortical expansion and cortical destruction amounted up to 1.23% and 6.77%, respectively. Inconsistent results were published in a Brazilian study, where the cortical expansion and cortical destruction was shown in 5.7% and 17.7%, respectively [98]. The abovementioned differences could be explained by the smaller sample size (300 *vs.* 480 individuals; 5585 *vs.* 8226 teeth) and lower participants age (12–70 years *vs.* 60–79 years). The discrepancies in the prevalence of AP between the current research and previous findings might be due to different evaluation methods: OPG [[16], [17], [18], [19], [20],[22], [23], [24], [25], [26], [27], [28], [29]], periapical radiographs [18,24,[30], [31], [32], [33], [34], [35], [36],^88^], or CBCT [[40], [41], [42],^88^], and clinical and radiographic examination [46,88]. Undoubtedly, on periapical radiograph and OPG the prevalence of AP may be underestimated [108]. Nevertheless, CBCT exhibits a higher diagnostic accuracy in dental pathosis compared to 2D radiography, because it is today the gold standard for imaging in the oral and maxillo-facial region [88].

### 4.9 Correlation between the root filling and apical periodontitis

Firstly, teeth with radiopaque material only in the pulp chamber and missed canals are one of the main reasons for AP [109]. In this finding the presence of root filling was associated with the AP, which was supported by other research [25,32,61,67,69,76,97]*.* Similarly to others [41], the present study reported that AP was found significantly more often in teeth with unprepared canals than in adequately prepared and obturated ones. Contrasting data were published in a study [86] that evaluated younger patients in Turkey, aged 15–72 years, and also a lower number of patients (242) using CBCT.

## 5 The limitations of the study

In this retrospective cohort study when only single observation period is evaluated, without considering the time at which root canal treatment was completed, it is not possible to understand if AP presented on CBCT was healing or growing lesion when compared. The study also actually lacks a clinical examination of patients, to compare radiological results with clinical aspects of examined teeth. Another limitation of the present study was the voxel sizes used (0.125–0.25), *viz.* the smaller voxel size (0.075) used the higher spatial resolution and more details are visible. The person conducting the study took part in the assessment of the study results, which may result in less objective and more positive results, which may be considered as a limitation of the study. Another limitation of this research was excluding edentulous individuals from the study, *i.e.,* it may be difficult to estimate the real oral health status of this elderly population. Additionally, the chi-square test used in the present study is sensitive to the sample size. When a very large sample is used, the tested relationships may appear to be significant even though they are not.

## 6 Conclusions

1. The number of teeth in elderly patients decreased, while the number of endodontically treated teeth increased with age.
2. More teeth were preserved in the mandible, the most common group of teeth were incisors. Molars were more frequently preserved in the maxilla, and premolars in the mandible.
3. The periapical lesions were more often detected in the maxilla in general, and in mandibular molars.
4. Inadequate prosthetic restorations were associated with increased prevalence of apical periodontitis.
5. Periapical lesions were found more often in root canal treated teeth, in teeth with the radiopaque material only in the pulp chamber in the single/multi-canal, and with missed root canals.
6. The over-filling was observed most frequently in the maxilla, in maxillary molars and in mandibular premolars, while the short-filling in maxillary and mandibular molars.
7. Periapical lesions were significantly most often observed in short-filled root canals than in other length criteria (overfilled, adequately, or lack of filling).

## Ethics statement

This retrospective cohort study was approved by The Ethics Committee of the Medical University of Lodz, with the approval number: RNN/333/15/KE.

All participants provided informed consent for the publication of their anonymized case details and images.

## Data availability statement

The datasets analysed during the current study are not publicly available but are available from the corresponding author on reasonable request.

## Funding

This study received no funding support.

## CRediT authorship contribution statement

**Krystyna Pietrzycka:** Writing – review & editing, Writing – original draft, Resources, Methodology, Investigation, Funding acquisition, Data curation, Conceptualization. **Mateusz Radwanski:** Writing – review & editing, Writing – original draft, Visualization, Investigation, Formal analysis, Data curation. **Jukka P. Matinlinna:** Writing – review & editing, Formal analysis. **Monika Lukomska-Szymanska:** Writing – review & editing, Writing – original draft, Visualization, Supervision, Project administration, Methodology, Investigation.

## Declaration of competing interest

The authors declare that they have no known competing financial interests or personal relationships that could have appeared to influence the work reported in this paper.

## References

[1] (2022). WHO. https://www.who.int/news-room/fact-sheets/detail/ageing-and-health.

[2] (2022). Eurostat.

[3] Kamińska-Gawryluk E., Wyszkowska D., Gabińska M., Romańska S. (2022). The situation of older people in Poland in 2021. http://Https://Stat.Gov.Pl/En/Topics/Older-People/Older-People/the-Situation-of-Older-People-in-Poland-in-2021,1,4.Html.

[4] Productivity Commission (2013).

[5] De Rossi S.S., Slaughter Y.A. (2007). Oral changes in older patients: a clinician's guide. Quintessence Int..

[6] Petersen P.E., Yamamoto T. (2005). Improving the oral health of older people: the approach of the WHO global oral health programme, community dent. Oral Epidemiol..

[7] Kandelman D., Petersen P.E., Ueda H. (2008). Oral health, general health, and quality of life in older people. Spec. Care Dent..

[8] Park H.-E., Song H.Y., Han K., Cho K.-H., Kim Y.-H. (2019). Number of remaining teeth and health-related quality of life: the Korean national health and nutrition examination survey 2010–2012. Health Qual. Life Outcome.

[9] Somsak K., Kaewplung O. (2016). The effects of the number of natural teeth and posterior occluding pairs on the oral health-related quality of life in elderly dental patients. Gerodontology.

[10] Chan A.K.Y., Tamrakar M., Jiang C.M., Lo E.C.M., Leung K.C.M., Chu C.-H. (2021). Common medical and dental problems of older adults: a narrative review. Geriatrics.

[11] Lamster I.B. (2016). Geriatric periodontology: how the need to care for the aging population can influence the future of the dental profession. Periodontol.

[12] Critén S., Andersson P., Renvert S., Götrick B., Berglund J.S., Bengtsson V.W. (2022). Oral health status among 60-year-old individuals born in 1941–1943 and 1954–1955 and 81-year-old individuals born in 1922–1924 and 1933–1934, respectively: a cross-sectional study. Clin. Oral Invest..

[13] Rodakowska E., Jamiolkowski J., Baginska J., Kaminska I., Gabiec K., Stachurska Z., Kondraciuk M., Dubatowka M., Kaminski K.A. (2022). Oral health–related quality of life and missing teeth in an adult population: a cross-sectional study from Poland. Int. J. Environ. Res. Publ. Health.

[14] Peres M.A., Macpherson L.M.D., Weyant R.J., Daly B., Venturelli R., Mathur M.R., Listl S., Celeste R.K., Guarnizo-Herreño C.C., Kearns C., Benzian H., Allison P., Watt R.G. (2019). Oral diseases: a global public health challenge. Lancet.

[15] Renvert S., Persson R.E., Persson G.R. (2013). Tooth loss and periodontitis in older individuals: results from the Swedish national study on aging and care. J. Periodontol..

[16] Chen C.Y., Hasselgren G., Serman N., Elkind M.S.V., Desvarieux M., Engebretson S.P. (2007). Prevalence and quality of endodontic treatment in the northern Manhattan elderly. J. Endod..

[17] Gulsahi K., Gulsahi A., Ungor M., Genc Y. (2008). Frequency of root-filled teeth and prevalence of apical periodontitis in an adult Turkish population. Int. Endod. J..

[18] Frisk F., Hugosson A., Kvist T. (2015). Is apical periodontitis in root filled teeth associated with the type of restoration?. Acta Odontol. Scand..

[19] Silnovic Z., Kvist T., Frisk F. (2022). Periapical status and technical quality in root canal filled teeth in a cross sectional study in Jönköping, Sweden. Acta Odontol. Scand..

[20] Närhi T.O., Leinonen K., Wolf J., Ainamo A. (2000). Longitudinal radiological study of the oral health parameters in an elderly Finnish population. Acta Odontol. Scand..

[21] Hollanda A.C.B., de Alencar A.H.G., Estrela C.R. de A., Bueno M.R., Estrela C. (2008). Prevalence of endodontically treated teeth in a Brazilian adult population. Braz. Dent. J..

[22] Sunay H., Tanalp J., Dikbas I., Bayirli G. (2007). Cross-sectional evaluation of the periapical status and quality of root canal treatment in a selected population of urban Turkish adults. Int. Endod. J..

[23] Loftus J.J., Keating A.P., McCartan B.E. (2005). Periapical status and quality of endodontic treatment in an adult Irish population. Int. Endod. J..

[24] Frisk F., Hugoson A., Hakeberg M. (2008). Technical quality of root fillings and periapical status in root filled teeth in Jönköping, Sweden. Int. Endod. J..

[25] Huumonen S., Suominen A.L., Vehkalahti M.M. (2017). Prevalence of apical periodontitis in root filled teeth: findings from a nationwide survey in Finland. Int. Endod. J..

[26] Timmerman A., Calache H., Parashos P. (2017). A cross sectional and longitudinal study of endodontic and periapical status in an Australian population. Aust. Dent. J..

[27] Bołtacz-Rzepkowska E., Pawlicka H. (2003). Radiographic features and outcome of root canal treatment carried out in the Łódź region of Poland. Int. Endod. J..

[28] Bołtacz-Rzepkowska E., Laszkiewicz J. (2005). [Endodontic treatment and periapical health in patients of the Institute of Dentistry in Lódź]. Przegl. Epidemiol..

[29] Kielbassa A.M., Frank W., Madaus T. (2017). Radiologic assessment of quality of root canal fillings and periapical status in an Austrian subpopulation – an observational study. PLoS One.

[30] Hebling E., Coutinho L.A., Ferraz C.C.R., Cunha F.L., Queluz D. de P. (2014). Periapical status and prevalence of endodontic treatment in institutionalized elderly. Braz. Dent. J..

[31] Costa T.H.R., Neto J.A. de F., de Oliveira A.E.F., e Maia M. de F.L., de Almeida A.L. (2014). Association between chronic apical periodontitis and coronary artery disease. J. Endod..

[32] Kirkevang L.-L., Hörsted-Bindslev P., Ørstavik D., Wenzel A. (2001). Frequency and distribution of endodontically treated teeth and apical periodontitis in an urban Danish population. Int. Endod. J..

[33] Georgopoulou M.K., Spanaki-Voreadi A.P., Pantazis N., Kontakiotis E.G. (2005). Frequency and distribution of root filled teeth and apical periodontitis in a Greek population. Int. Endod. J..

[34] Haereid M.K., Stangvaltaite-Mouhat L., Ansteinsson V., Mdala I., Ørstavik D. (2022). Periapical status transitions in teeth with posts versus without posts: a retrospective longitudinal radiographic study. Acta Odontol. Scand..

[35] Diep M.T., Hove L.H., Ørstavik D., Skudutyte-Rysstad R., Sødal A.T.T., Sunde P.T. (2022). Periapical and endodontic status among 65-year-old Oslo-citizens. BMC Oral Health.

[36] Imfeld T.N. (1991). Prevalence and quality of endodontic treatment in an elderly urban population of Switzerland. J. Endod..

[37] Davies A., Patel S., Foschi F., Andiappan M., Mitchell P.J., Mannocci F. (2016). The detection of periapical pathoses using digital periapical radiography and cone beam computed tomography in endodontically retreated teeth - part 2: a 1 year post-treatment follow-up. Int. Endod. J..

[38] Patel S., Dawood A., Whaites E., Pitt Ford T. (2009). New dimensions in endodontic imaging: part 1. Conventional and alternative radiographic systems. Int. Endod. J..

[39] Gliga A., Imre M., Grandini S., Marruganti C., Gaeta C., Bodnar D., Dimitriu B.A., Foschi F. (2023). The limitations of periapical X-ray assessment in endodontic diagnosis—a systematic review. J. Clin. Med..

[40] Meirinhos J., Martins J.N.R., Pereira B., Baruwa A., Gouveia J., Quaresma S.A., Monroe A., Ginjeira A. (2020). Prevalence of apical periodontitis and its association with previous root canal treatment, root canal filling length and type of coronal restoration – a cross-sectional study. Int. Endod. J..

[41] Bürklein S., Schäfer E., Jöhren H.P., Donnermeyer D. (2020). Quality of root canal fillings and prevalence of apical radiolucencies in a German population: a CBCT analysis. Clin. Oral Invest..

[42] Van der Veken D., Curvers F., Fieuws S., Lambrechts P. (2017). Prevalence of apical periodontitis and root filled teeth in a Belgian subpopulation found on CBCT images. Int. Endod. J..

[43] Orstavik D., Qvist V., Stoltze K. (2004). A multivariate analysis of the outcome of endodontic treatment. Eur. J. Oral Sci..

[44] De Moor R.J.G., Hommez G.M.G., De Boever J.G., Delme K.I.M., Martens G.E.I. (2000). Periapical health related to the quality of root canal treatment in a Belgian population. Int. Endod. J..

[45] Razdan A., Jungnickel L., Schropp L., Væth M., Kirkevang L.L. (2022). Trends of endodontic and periapical status in adult Danish populations from 1997 to 2009: a repeated cross-sectional study. Int. Endod. J..

[46] Connert T., Truckenmüller M., ElAyouti A., Eggmann F., Krastl G., Löst C., Weiger R. (2019). Changes in periapical status, quality of root fillings and estimated endodontic treatment need in a similar urban German population 20 years later. Clin. Oral Invest..

[47] Kayahan M.B., Malkondu Ö., Canpolat C., Kaptan F., Bayırlı G., Kazazoglu E. (2008). Periapical health related to the type of coronal restorations and quality of root canal fillings in a Turkish subpopulation. Oral Surg. Oral Med. Oral Pathol. Oral Radiol. Endod..

[48] López-López J., Jané-Salas E., Estrugo-Devesa A., Velasco-Ortega E., Martín-González J., Segura-Egea J.J. (2011). Periapical and endodontic status of type 2 diabetic patients in Catalonia, Spain: a cross-sectional study. J. Endod..

[49] Hussein F.E., Liew A.K.C., Ramlee R.A., Abdullah D., Chong B.S. (2016). Factors associated with apical periodontitis: a multilevel analysis. J. Endod..

[50] Al-Omari M.A., Hazaa A., Haddad F. (2011). Frequency and distribution of root filled teeth and apical periodontitis in a Jordanian subpopulation. Oral Surg. Oral Med. Oral Pathol. Oral Radiol. Endod..

[51] Gomes A.C., Nejaim Y., Silva A.I.V., Haiter-Neto F., Cohenca N., Zaia A.A., Silva E.J.N.L. (2015). Influence of endodontic treatment and coronal restoration on status of periapical tissues: a cone-beam computed tomographic study. J. Endod..

[52] Estrela C., Bueno M.R., Azevedo B.C., Azevedo J.R., Pécora J.D. (2008). A new periapical index based on cone beam computed tomography. J. Endod..

[53] Lemagner F., Maret D., Peters O.A., Arias A., Coudrais E., Georgelin-Gurgel M. (2015). Prevalence of apical bone defects and evaluation of associated factors detected with cone-beam computed tomographic images. J. Endod..

[54] https://www.checkmarket.com/kb/calculate-optimal-sample-size-survey/, (n.d.).

[55] Vasconcelos K.F., Nicolielo L.F.P., Nascimento M.C., Haiter-Neto F., Bóscolo F.N., Van Dessel J., EzEldeen M., Lambrichts I., Jacobs R. (2015). Artefact expression associated with several cone-beam computed tomographic machines when imaging root filled teeth. Int. Endod. J..

[56] Rodrigues C.T., Jacobs R., Vasconcelos K.F., Lambrechts P., Rubira-Bullen I.R.F., Gaêta-Araujo H., Oliveira-Santos C., Duarte M.A.H. (2021). Influence of CBCT-based volumetric distortion and beam hardening artefacts on the assessment of root canal filling quality in isthmus-containing molars. Dentomaxillofacial Radiol..

[57] Abdinian M., Moshkforoush S., Hemati H., Soltani P., Moshkforoushan M., Spagnuolo G. (2020). Comparison of cone beam computed tomography and digital radiography in detecting separated endodontic files and strip perforation. Appl. Sci..

[58] Schulze R., Heil U., Groβ D., Bruellmann D., Dranischnikow E., Schwanecke U., Schoemer E. (2011). Artefacts in CBCT: a review. Dentomaxillofacial Radiol..

[59] Madadizadeh F., Ghafari H., Bahariniya S. (2023). Kappa statistics a method of measuring agreement in dental examinations. http://Https://Www.Researchsquare.Com/Article/Rs-2535291/V1.

[60] Müller F., Naharro M., Carlsson G.E. (2007). What are the prevalence and incidence of tooth loss in the adult and elderly population in Europe?. Clin. Oral Implants Res..

[61] Di Filippo G., Sidhu S.K., Chong B.S. (2014). Apical periodontitis and the technical quality of root canal treatment in an adult sub-population in London. Br. Dent. J..

[62] Hamedy R., Shakiba B., Pak J.G., Barbizam J.V., Ogawa R.S., White S.N. (2016). Prevalence of root canal treatment and periapical radiolucency in elders: a systematic review. Gerodontology.

[63] Frisk F., Merdad K., Reit C., Hugoson A., Birkhed D. (2011). Root-filled teeth and recurrent caries—a study of three repeated cross-sectional samples from the city of Jönköping, Sweden. Acta Odontol. Scand..

[64] Jakovljevic A., Nikolic N., Jacimovic J., Pavlovic O., Milicic B., Beljic-Ivanovic K., Miletic M., Andric M., Milasin J. (2020). Prevalence of apical periodontitis and conventional nonsurgical root canal treatment in general adult population: an updated systematic review and meta-analysis of cross-sectional studies published between 2012 and 2020. J. Endod..

[65] Lõpez-Lõpez J., Jané-Salas E., Estrugo-Devesa A., Castellanos-Cosano L., Martín-González J., Velasco-Ortega E., Segura-Egea J.J. (2012). Frequency and distribution of root-filled teeth and apical periodontitis in an adult population of Barcelona, Spain. Int. Dent. J..

[66] Moreno J.O., Alves F.R.F., Gonçalves L.S., Martinez A.M., Rôças I.N., Siqueira J.F. (2013). Periradicular status and quality of root canal fillings and coronal restorations in an urban colombian population. J. Endod..

[67] Vengerfeldt V., Mändar R., Nguyen M.S., Saukas S., Saag M. (2017). Apical periodontitis in southern Estonian population: prevalence and associations with quality of root canal fillings and coronal restorations. BMC Oral Health.

[68] Dutta A., Smith-Jack F., Saunders W.P. (2014). Prevalence of periradicular periodontitis in a Scottish subpopulation found on CBCT images. Int. Endod. J..

[69] Liang Y.-H., Li G., Shemesh H., Wesselink P.R., Wu M.-K. (2012). The association between complete absence of post-treatment periapical lesion and quality of root canal filling. Clin. Oral Invest..

[70] Kvist T., Rydin E., Reit C. (1989). The relative frequency of periapical lesions in teethwith root canal-retained posts. J. Endod..

[71] Skudutyte-Rysstad R., Eriksen H.M. (2006). Endodontic status amongst 35-year-old Oslo citizens and changes over a 30-year period. Int. Endod. J..

[72] Özbaş H., Aşci S., Aydin Y. (2011). Examination of the prevalence of periapical lesions and technical qulity of endodontic treatment in a Turkish subpopulation, Oral Surgery. Oral Med. Oral Pathol. Oral Radiol. Endodontology.

[73] Hugoson A., Koch G., Göthberg C., Helkimo A.N., Lundin S.-A., Norderyd O., Sjödin B., Sondell K. (2005). Oral health of individuals aged 3-80 years in Jönköping, Sweden during 30 years (1973-2003). II. Review of clinical and radiographic findings. Swed. Dent. J..

[74] León-López M., Cabanillas-Balsera D., Martín-González J., Montero-Miralles P., Saúco-Márquez J.J., Segura-Egea J.J. (2022). Prevalence of root canal treatment worldwide: a systematic review and meta-analysis. Int. Endod. J..

[75] Tsuneishi M., Yamamoto T., Yamanaka R., Tamaki N., Sakamoto T., Tsuji K., Watanabe T. (2005). Radiographic evaluation of periapical status and prevalence of endodontic treatment in an adult Japanese population, Oral Surgery. Oral Med. Oral Pathol. Oral Radiol. Endodontology.

[76] Ahmed I., Ali R.W., Mudawi A.M. (2017). Prevalence of apical periodontitis and frequency of root-filled teeth in an adult Sudanese population. Clin. Exp. Dent. Res..

[77] Kabak Y., Abbott P.V. (2005). Prevalence of apical periodontitis and the quality of endodontic treatment in an adult Belarusian population. Int. Endod. J..

[78] Siqueira J.F., Rôças I.N., Alves F.R.F., Campos L.C. (2005). Periradicular status related to the quality of coronal restorations and root canal fillings in a Brazilian population, Oral Surgery. Oral Med. Oral Pathol. Oral Radiol. Endodontology.

[79] Dawson V., Petersson K., Wolf E., Akerman S. (2014). Periapical status of non-root-filled teeth with resin composite, amalgam, or full crown restorations: a cross-sectional study of a Swedish adult population. J. Endod..

[80] Ainamo A., Soikkonen K., Wolf J., Siukosaari P., Erkinjuntti T., Tilvis R., Valvanne J. (1994). Dental radiographic findings in the elderly in Helsinki, Finland. Acta Odontol. Scand..

[81] Frisk F., Hakeberg M. (2006). Socio-economic risk indicators for apical periodontitis. Acta Odontol. Scand..

[82] Ferreira R.C., de Magalhães C.S., Moreira A.N. (2008). Tooth loss, denture wearing and associated factors among an elderly institutionalised Brazilian population. Gerodontology.

[83] El Ouarti I., Chala S., Sakout M., Abdallaoui F. (2021). Prevalence and risk factors of Apical periodontitis in endodontically treated teeth: cross-sectional study in an Adult Moroccan subpopulation. BMC Oral Health.

[84] Virtanen E., Nurmi T., Söder P.-Ö., Airila-Månsson S., Söder B., Meurman J.H. (2017). Apical periodontitis associates with cardiovascular diseases: a cross-sectional study from Sweden. BMC Oral Health.

[85] Lupi-Pegurier L., Bertrand M.-F., Muller-Bolla M., Rocca J.P., Bolla M. (2002). Periapical status, prevalence and quality of endodontic treatment in an adult French population. Int. Endod. J..

[86] Nur B.G., Ok E., Altunsoy M., Ağlarci O.S., Çolak M., Güngör E. (2014). Evaluation of technical quality and periapical health of root-filled teeth by using cone-beam CT. J. Appl. Oral Sci..

[87] Ali A., Mahdee A., Fadhil N., Shihab D. (2022). Prevalence of periapical lesions in non-endodontically and endodontically treated teeth in an urban Iraqi adult subpopulation: a retrospective CBCT analysis. J. Clin. Exp. Dent..

[88] Keerthana G., Singh N., Yadav R., Duhan J., Tewari S., Gupta A., Sangwan P., Mittal S. (2021). Comparative analysis of the accuracy of periapical radiography and cone-beam computed tomography for diagnosing complex endodontic pathoses using a gold standard reference – a prospective clinical study. Int. Endod. J..

[89] Persic Bukmir R., Paljevic E., Vidas J., Glazar I., Pezelj-Ribaric S., Brekalo Prso I. (2022). Is coronal restoration a predictor of posttreatment apical periodontitis?. Eur. J. Dermatol..

[90] Eckerbom M., Magnusson T., Martinsson T. (1991). Prevalence of apical periodontitis, crowned teeth and teeth with posts in a Swedish population. Dent. Traumatol..

[91] Boucher Y., Matossian L., Rilliard F., Machtou P. (2002). Radiographic evaluation of the prevalence and technical quality of root canal treatment in a French subpopulation. Int. Endod. J..

[92] da Luz-Silva G., Vetromilla B.M., Pereira-Cenci T. (2022). Influence of post type on periapical status: a prospective study in a Brazilian population. Clin. Oral Invest..

[93] Hommez G.M.G., Coppens C.R.M., De Moor R.J.G. (2002). Periapical health related to the quality of coronal restorations and root fillings. Int. Endod. J..

[94] Estrela C., Bueno M.R., Porto O.C.L., Rodrigues C.D., Pécora J.D. (2009). Influence of intracanal post on apical periodontitis identified by cone-beam computed tomography. Braz. Dent. J..

[95] Tibúrcio-Machado C.S., Michelon C., Zanatta F.B., Gomes M.S., Marin J.A., Bier C.A. (2021). The global prevalence of apical periodontitis: a systematic review and meta-analysis. Int. Endod. J..

[96] Mujawar A., Hegde V., Srilatha S. (2021). A retrospective three-dimensional assessment of the prevalence of apical periodontitis and quality of root canal treatment in Mid-West Indian population. J. Conserv. Dent..

[97] Peters L.B., Lindeboom J.A., Elst M.E., Wesselink P.R. (2011). Prevalence of apical periodontitis relative to endodontic treatment in an adult Dutch population: a repeated cross-sectional study, Oral Surgery. Oral Med. Oral Pathol. Oral Radiol. Endodontology.

[98] Da Silva Ramos Fernandes L.M.P., Ordinola-Zapata R., Hungaro Duarte M.A., Alvares Capelozza A.L. (2013). Prevalence of apical periodontitis detected in cone beam CT images of a Brazilian subpopulation. Dentomaxillofacial Radiol..

[99] Meirinhos J., Martins J.N.R., Pereira B., Baruwa A.O., Ginjeira A. (2021). Prevalence of lateral radiolucency, apical root resorption and periapical lesions in Portuguese patients: a CBCT cross-sectional study with a worldwide overview. Eur. Endod. J..

[100] Baruwa A.O., Martins J.N.R., Meirinhos J., Pereira B., Gouveia J., Quaresma S.A., Monroe A., Ginjeira A. (2020). The influence of missed canals on the prevalence of periapical lesions in endodontically treated teeth: a cross-sectional study. J. Endod..

[101] Johnstone M., Parashos P. (2015). Endodontics and the ageing patient. Aust. Dent. J..

[102] Karabucak B., Bunes A., Chehoud C., Kohli M.R., Setzer F. (2016). Prevalence of apical periodontitis in endodontically treated premolars and molars with untreated canal: a cone-beam computed tomography study. J. Endod..

[103] Aysal Z., Demirturk Kocasarac H., Orhan K., Helvacioglu-Yigit D. (2022). Radiological assessment of prevalance and quality of periapical status of endodontic treatments. Med. Sci. Monit..

[104] Karteva T., Manchorova-Veleva N.A., Karteva E., Keskinova D., Kanazirska P., Jordanov G., Vladimirov S. (2021). Quality of endodontic treatment and prevalence of apical radiolucencies in a Bulgarian subpopulation: a CBCT analysis. Folia Med. (Plovdiv)..

[105] Cakici E., Yildirim E., Cakici F., Erdogan A. (2016). Assessment of periapical health, quality of root canal filling, and coronal restoration by using cone-beam computed tomography, Niger. J. Clin. Pract.

[106] Fernández R., Cardona J.A., Cadavid D., Álvarez L.G., Restrepo F.A. (2017). Survival of endodontically treated roots/teeth based on periapical health and retention: a 10-year retrospective cohort study. J. Endod..

[107] Alves dos Santos G.N., Faria-e-Silva A.L., Ribeiro V.L., Pelozo L.L., Candemil A.P., Oliveira M.L., Lopes-Olhê F.C., Mazzi-Chaves J.F., Sousa-Neto M.D. (2022). Is the quality of root canal filling obtained by cone-beam computed tomography associated with periapical lesions? A systematic review and meta-analysis. Clin. Oral Invest..

[108] Estrela C., Bueno M.R., Leles C.R., Azevedo B., Azevedo J.R. (2008). Accuracy of cone beam computed tomography and panoramic and periapical radiography for detection of apical periodontitis. J. Endod..

[109] Cantatore G., Berutti E., Castellucci A. (2006). Missed anatomy: frequency and clinical impact. Endod. Top..

