# Supplementary material for: The prevalence of root canal treatment, periapical status, and coronal restorations in elderly patients in the Polish population
Source: Heliyon. 2024 Aug 21;10(17):e35584. doi: 10.1016/j.heliyon.2024.e35584 (PMC11408157; doi:10.1016/j.heliyon.2024.e35584)
Supplement: Multimedia component 1 [file mmc1.docx]

A total of 480 individuals, 220 (45.83%) men and 260 (54.17%) women, were included into the study. The average age of the study population was 67.72 years, ranged between 60 to 79 years (men: 67.35 years, and women: 68.02 years). Moreover, 57.70% patients were in age of 60-69, and 42.30% individuals aged 70 and over.
