# Supplementary material for: The prevalence of root canal treatment, periapical status, and coronal restorations in elderly patients in the Polish population
Source: Heliyon. 2024 Aug 21;10(17):e35584. doi: 10.1016/j.heliyon.2024.e35584 (PMC11408157; doi:10.1016/j.heliyon.2024.e35584)
Supplement: Multimedia component 2 [file mmc2.docx]

There were no statistically significant differences between gender and age and the presence of apical periodontitis (p >0.05).
