# Supplementary material for: The prevalence of root canal treatment, periapical status, and coronal restorations in elderly patients in the Polish population
Source: Heliyon. 2024 Aug 21;10(17):e35584. doi: 10.1016/j.heliyon.2024.e35584 (PMC11408157; doi:10.1016/j.heliyon.2024.e35584)
Supplement: Multimedia component 3 [file mmc3.docx]

There is a no statistically significant difference in the presence of AP between the genders. The present study confirms previous reports [32,35,84], showing lack of significant difference in the presence of AP between the genders. However, other studies reported higher incidence of AP in men [24,25,28,51] or in women [85].

In this and in previous studies, AP in root canal filled teeth was not related with the age [19,25,29], which is not supported by others [23,28,73,75]. These divergences could be attributed to the differences in methodological concept, namely number of participants and groups in different age range.
