# Supplementary material for: The prevalence of root canal treatment, periapical status, and coronal restorations in elderly patients in the Polish population
Source: Heliyon. 2024 Aug 21;10(17):e35584. doi: 10.1016/j.heliyon.2024.e35584 (PMC11408157; doi:10.1016/j.heliyon.2024.e35584)
Supplement: Multimedia component 4 [file mmc4.docx]

The mean number of teeth decrease with age similarly as in other studies [27,28,60–62,73,82,86]. Moreover, the average number of preserved teeth in the investigated population amounted up to 17.14 teeth (1-32 teeth). This result corresponds with data for Polish subpopulation (17.1) [28], and with US subpopulation [16]. Interestingly, higher number of teeth was observed in Norwegian (26) [35], Swedish (18-23) [24,73,81], Danish (22.7-26) [32,45], English subpopulations (18) [61] and in Estonian adult population aged 35.5±19.2 years (27.7) [67]. On the contrary, lower mean number of the teeth per subject was determined in Finnish subpopulation (13.2) [20] and institutionalized Brazilian elderly individuals (11.6) [30,82]. The average number of teeth in women in the present study was higher than in men, opposite data were earlier presented [28].

Additionally, more teeth were preserved in the mandible (average 9.26 teeth) than in the maxilla (average 7.88 teeth), what was consistent with Norwegian [35] and German adult population (50.21±12.45 years) [41]. Similarly to this research mandibular molars were often missing in Estonian population (mean 35.5±19.2 years) [67]. The most common teeth in both the maxilla and mandible were the incisors similarly to other studies [33,35,40,68]. Moreover, molars were more frequently preserved in the maxilla while premolars in mandible, what is supported by others [35,68]. These results can be explained that due to socioeconomic status of this study group; lower income then younger adults (professionally active), therefore elderly cannot afford expensive root canal treatment and prosthodontic restoration and decide to extract the tooth. Another explanation is the complex anatomy of maxillary molars, so extraction might be the prevailing treatment of choice.
