# Supplementary material for: The prevalence of root canal treatment, periapical status, and coronal restorations in elderly patients in the Polish population
Source: Heliyon. 2024 Aug 21;10(17):e35584. doi: 10.1016/j.heliyon.2024.e35584 (PMC11408157; doi:10.1016/j.heliyon.2024.e35584)
Supplement: Multimedia component 5 [file mmc5.docx]

The present study revealed statistically significant correlation between the age of the patients and the number of preserved teeth. With age, the number of teeth in patients decreases what was confirmed in previous reports [20,21,23,27,28,32,73,75,76,80]. The prevalence of endodontically treated teeth increased with age, what was corroborated in the literature [28,32,75,76,84] and in the present study. Contrasting data was presented in Greek, where this number decreased with age in patients over 60 years [33]. A total of 88.54% of patients in present study had at least one endodontically treated tooth, this corresponds with the finding from Japan 87% [75]. Lower results were found in USA (38.8%) [16], Finnish (68-78%) [25,80] and Swiss subpopulations (77.6%) [36]. However, higher prevalence of RCT was determined (92%) for individuals over 70 years in the Swedish elderly patient [87].

A total of 22.56% teeth were endodontically treated in current paper, similarly to the previous research [80,88]. However, higher prevalence was found in Japan (30%) [75] and in Brazil (32.7%) [21], but lower in USA (5.1%) [16], Greek (9.7%) [33], Denmark (12.8%) [32], Sweden (17.8-19.4%) [24,73] and Switzerland (20.3%) [36].
