# Supplementary material for: The prevalence of root canal treatment, periapical status, and coronal restorations in elderly patients in the Polish population
Source: Heliyon. 2024 Aug 21;10(17):e35584. doi: 10.1016/j.heliyon.2024.e35584 (PMC11408157; doi:10.1016/j.heliyon.2024.e35584)
Supplement: Multimedia component 6 [file mmc6.docx]

The authors evaluated 8226 teeth, of which 22.56% were endodontically treated. In this retrospective study, the prevalence of teeth with RCT was gender independent, confirming what has been previously reported [32,41,76,100,101], and what was inconsistent with some other results [21,67]. The prevalence of teeth with RCT in this research (22.56%) was similar to others findings [18,20,21,102], and higher than earlier published (4.8-12.8%) [16,32,33,40,41,67]. The current report is in disagreement with some other studies showing higher prevalence of root canal treatments - 23-33.6% [21,87,88]. These discrepancies might be due to the higher sample of individuals [32] or a lower number of teeth [16], or differences in the characteristics of population [21,40,41].
